# Robotic surgery in France: insights from a national administrative database on utilization, access, and efficiency

**DOI:** 10.1007/s11701-026-03188-w

**Published:** 2026-02-23

**Authors:** G. Saiydoun, G. Micicoï, J.-C. Couffinhal, H. Johanet, A.-C. Benhamou, P. Leprince, A. Delafontaine

**Affiliations:** 1 Department of Cardiac and Thoracic Surgery, Pitié-Salpêtrière University Hospital, Sorbonne University, APHP, Paris, France; 2 National Academy of Surgery, Paris, France; 3 University Institute of Locomotor and Sports (iULS), Pasteur II Hospital, 30, Voie Romaine, 06000 Nice, France; 4 ICARE Unit, Inserm U1091, Côte d’Azur University, Nice, France; 5 CIAMS Laboratory, Université Paris-Saclay, 91404 Orsay, France

**Keywords:** Robotic-assisted surgery, Minimally invasive surgery, National registry, Regional disparities, Surgery 4.0

## Abstract

Robotic-assisted surgery has become an integral component of minimally invasive surgery, yet its nationwide diffusion and real-world impact in France have only recently become measurable following the implementation of mandatory procedural traceability. We conducted a nationwide, retrospective, cross-sectional study. All adult patients undergoing selected urologic, digestive, gynecologic, and thoracic procedures between January 2021 and December 2022 were included. Surgical approaches were classified as robotic-assisted, laparoscopic, or open. Primary outcomes were national volumes, robotic penetration rates, and regional distribution. Secondary outcomes included length of stay, intensive care unit admission, and 30- and 90-day readmission rates. A total of 58 232 robotic-assisted procedures were identified, increasing from 27 011 in 2021 to 31,221 in 2022 (+ 15.6%), and accounting for 15.6% of minimally invasive procedures. Urology predominated (61%), followed by digestive (17%), gynecologic (15%), and thoracic surgery (7%). Robotic penetration reached 54.9% in urology but remained below 15% in other specialties. Marked regional disparities were observed, with Île-de-France accounting for over 27% of all robotic procedures, while several regions reported minimal or no activity. Compared with laparoscopic and open surgery, robotic-assisted procedures were associated with shorter length of stay (4.2 vs. 5.7 and 7.9 days, respectively), lower intensive care unit admission rates (6.3% vs. 9.7% and 14.1%), and reduced 30-day readmissions (4.8% vs. 5.6% and 6.9%). Robotic-assisted surgery in France is expanding and associated with improved early postoperative outcomes, but its adoption remains highly uneven, highlighting the need for coordinated national planning.

## Introduction

Over the past two decades, robotic-assisted surgery (RAS) has profoundly transformed surgical practice, marking a new stage in the evolution of minimally invasive surgery. Following the laparoscopic revolution of the 1990s, robotic systems introduced enhanced three-dimensional vision, tremor filtration, and increased precision of motion, enabling complex interventions to be performed with greater safety and reproducibility. These technological advances have contributed to shorter hospital stays, reduced postoperative pain, and faster recovery compared to conventional open approaches [1–3].

Despite its proven technical advantages, the deployment of robotic-assisted surgery in France has long remained untraceable in national databases. Before 2020, information regarding the diffusion of robotics was mainly derived from industry reports, often limited to the number of installed platforms or estimated procedures, with little epidemiological or clinical granularity [4]. In contrast, several countries such as the United States have used nationwide databases to continuously monitor surgical activity and outcomes. Wright et al. analysed more than 7.4 million inpatient hysterectomies performed between 1998 and 2010 in the Nationwide Inpatient Sample, showing a 36.4% decrease in annual case volume over this period [5].

In several high-income countries, nationwide surgical data infrastructures support continuous monitoring of surgical activity and outcomes. In the United Kingdom, NHS Digital and national clinical audit programs such as the National Hip Fracture Database provide routine nationwide surveillance of surgical care and outcomes. Similarly, Scandinavian countries have developed comprehensive national patient registries and clinical quality registries; in Sweden and Denmark, these systems enable longitudinal assessment of a wide range of surgical procedures and postoperative outcomes at the population level. In the United States, analyses of the Nationwide Inpatient Sample have likewise informed long-term trends in operative practice, including changes in surgical volume and approach [6, 7]. In contrast, in France, although the French Medical Information System Program (PMSI) has long captured hospital discharge data, the absence of specific French Common Classification of Medical Procedures (CCAM) procedure coding for robotic surgery before 2020 limited the ability to accurately track robotic surgical activity at a national level.

Since March 2020, France has introduced mandatory traceability for specific robotic procedures according to data from the PMSI, the national database that records standardized hospital discharge summaries in France, through extensions according to procedure codes from the CCAM, the national coding system for medical acts in France. This initiative provides, for the first time, the opportunity to quantify national robotic activity and analyze regional variations across surgical specialties. Such monitoring is crucial in a context of ongoing transformation of the healthcare system toward digitalization and personalized medicine, the so-called “Medicine 5P” (predictive, preventive, personalized, participative, and proven-effective) [8, 9].

The benefits of robotic-assisted surgery for soft tissues are now widely acknowledged. The French National Health Insurance Fund (CNAM) emphasized in its 2020 “the hospital’s Expenses and Revenues report” report that robotic assistance improves the surgeon’s vision and dexterity, facilitates access to deep anatomical regions, and enhances intraoperative comfort [10]. Similarly, a large systematic review and meta-analysis demonstrated that robotic-assisted surgery is associated with reduced intraoperative blood loss, shorter hospital length of stay, and lower rates of postoperative complications, readmissions, and mortality compared with open surgery, across multiple oncologic procedures [11]. However, the expansion of RAS also raises concerns regarding cost-effectiveness, equitable access, and the lack of large-scale real-world data to confirm its long-term impact on outcomes and healthcare organization [12, 13].

In France, robotic technology was initially adopted in urology, particularly for radical prostatectomy, before extending to gynecologic, digestive, and thoracic surgery. The number of robotic systems increased from 135 in 2017 to approximately 260 in 2023 [14]. Nevertheless, this diffusion remains heterogeneous, with significant regional and institutional disparities.

Understanding these variations is essential to ensure the relevance, quality, and efficiency of surgical care nationwide. Although robotic surgery is rapidly expanding, very few studies have evaluated its large-scale trends at the population level, and even fewer have investigated potential territorial disparities in its adoption and use. In the digital era of “Surgery 4.0”, robotics serves not only as a surgical tool but as a connected platform integrated into a broader ecosystem of diagnostic, therapeutic, and organizational data [15]. This paradigm shift therefore calls for a reassessment of healthcare policies, funding mechanisms, and training frameworks to guarantee that innovation benefits patients equitably across all regions. The present study, conducted by the French National Academy of Surgery in collaboration with the PMSI database, aims to provide the first consolidated national overview of robotic-assisted soft tissue surgery in France. Its objectives are threefold: to describe national activity and trends from 2021 to 2022; to assess regional disparities and access to robotic and other minimally invasive approaches; and to evaluate early real-world outcomes in terms of length of stay, intensive care use, and readmission rates. Beyond a quantitative analysis, this work seeks to inform national health strategy and to determine whether France is adequately prepared to embrace the challenges of Surgery 4.0 in the era of 5P Medicine.

## Methods

### Study design

This nationwide, retrospective, cross-sectional study was conducted using data from the PMSI. The analysis covered the years 2021 and 2022, corresponding to the first complete period after the implementation of specific traceability codes for robotic-assisted procedures in CCAM.

The study was carried out under the coordination of the French National Academy of Surgery in collaboration with the CNAM through PMSI.

**Fig. 1:**
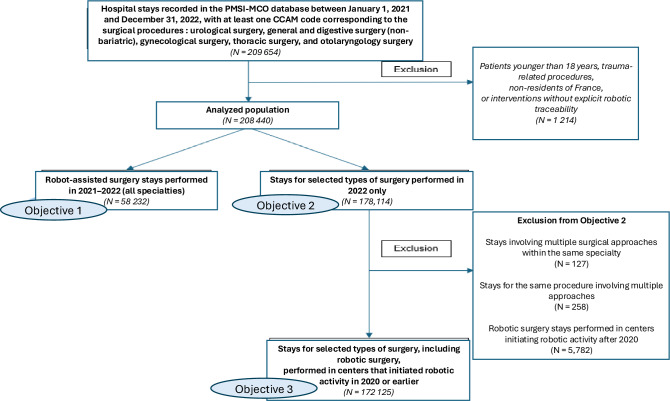
Flowchart of study population selection and analytical objectives. The study included hospital stays recorded in the PMSI-MCO database between January 1, 2021 and December 31, 2022 for urological surgery, general and digestive surgery (non-bariatric), gynecological surgery, thoracic surgery, and otolaryngology surgery, identified using predefined CCAM codes. Trauma-related procedures, procedures without explicit robotic traceability, patients younger than 18 years, and non-residents of France were excluded. Objective 1 included all robot-assisted surgery stays performed in 2021–2022. Objective 2 focused on stays for selected surgical procedures performed in 2022 only, regardless of surgical approach. Objective 3 was derived from Objective 2 after excluding stays involving multiple surgical approaches and robotic procedures performed in centers initiating robotic activity after 2020, in order to ensure stabilized robotic activity and limit learning-curve effects

The study population was analyzed according to three predefined objectives. Pediatric cases, trauma-related procedures, non-residents of France, and interventions without explicit robotic traceability were excluded. Because these exclusion criteria were not mutually exclusive and could overlap within the same hospital stay, they were applied jointly, resulting in a total of 1,214 excluded hospital stays (Fig. 1).

Objective 1 included all robot-assisted surgery stays performed in 2021 and 2022 across all eligible surgical specialties. Objective 2 focused on selected types of surgery performed in 2022 only, including robotic, laparoscopic, and open approaches, allowing cross-sectional comparison between surgical techniques within the same calendar year. Objective 3 was derived from Objective 2 after excluding stays involving multiple surgical approaches and robotic procedures performed in centers that initiated robotic activity after 2020, in order to reduce heterogeneity related to early adoption and learning-curve effects.

### Data source

The PMSI database exhaustively records all hospitalizations in public and private healthcare facilities across France, including Medicine, Surgery and Obstetrics (MSO) stays. Each hospitalization file includes demographic characteristics (age, sex, postal code), primary and secondary diagnoses coded using the *International Classification of Diseases, 10th Revision* (ICD-10), and medical or surgical procedures coded with the CCAM classification.

Since March 2020, the PMSI has incorporated an explicit identification of robotic-assisted procedures through additional CCAM codes (suffix “K” or equivalent) for eligible soft-tissue surgical acts. This new traceability enabled the extraction of all robotic procedures performed nationwide and their comparison with equivalent laparoscopic or open approaches.

### Ethical and regulatory considerations

All analyses were conducted in compliance with French data protection regulations. The PMSI data used were fully anonymized and aggregated prior to analysis. According to French law (French Public Health Code, Articles L. 6113–7 and R. 6113–9), studies using anonymized PMSI data for research and public health purposes do not require individual patient consent or ethics committee approval.

The study adhered to the principles of the General Data Protection Regulation (GDPR, EU 2016/679) and followed the STROBE (Strengthening the Reporting of Observational Studies in Epidemiology) recommendations for cross-sectional studies.

### Study population

All adult patients (≥ 18 years old) hospitalized between January 1, 2021, and December 31, 2022, for selected soft-tissue procedures were included. Surgical categories analyzed were:

- Urology (e.g., radical prostatectomy, partial nephrectomy, cystectomy);
- Digestive surgery (e.g., colectomy, rectal resection);
- Gynecology (e.g., hysterectomy, myomectomy);
- Thoracic surgery (e.g., lobectomy, thymectomy).

Procedures were categorized into three groups according to the approach: robotic-assisted, conventional laparoscopic/thoracoscopic, and open surgery. Pediatric cases, trauma-related procedures, and interventions without explicit traceability were excluded.

### Variables and outcomes

For each hospitalization, the following data were extracted:

- Patient demographics (age, sex);
- Type of hospital (Public University Hospital; General Hospital; ESPIC: Private Non-Profit Hospital of Collective Interest; Private for-profit hospital);
- Surgical specialty and anatomical site;
- Surgical approach (robotic, laparoscopic, open);
- Primary outcomes: total number of procedures, robotic penetration rate (i.e. defined as the proportion of robotic procedures among all coded interventions for a given indication), and regional distribution per 100,000 inhabitants.
- Secondary outcomes**:** hospital length of stay (LOS), intensive care unit (ICU) admission rate, 30- and 90-day readmission rates, and conversion to open surgery where applicable.

Regional data were linked to the patient’s residence to map national disparities across the 13 metropolitan regions and overseas departments (DROM-COM).

### Statistical analysis

Descriptive statistics were used to summarize patient and procedural characteristics. Quantitative variables are expressed as mean ± standard deviation (SD) or median with interquartile range (IQR), depending on normality. Qualitative variables are reported as frequencies and percentages.

Comparisons between groups (robotic vs laparoscopic vs open) were performed using the student’s t-test or the Wilcoxon rank-sum test for continuous variables, and the Chi-square test for categorical variables. A p-value < 0.05 was considered statistically significant.

Geographical distribution maps and choropleth visualizations of robotic penetration rates were generated using *SAS® v9.4* (SAS Institute Inc., Cary, NC, USA) and *QGIS v3.22* (Open Source Geospatial Foundation).

## Results

### National activity and temporal trends

Between January 2021 and December 2022, a total of 58 912 hospital stays involving robotic-assisted soft-tissue surgery were recorded in the national PMSI database, including 27 011 in 2021 and 31 221 in 2022 (Fig. 1), representing a 15.6% year-to-year increase. Over the same period, robotic procedures accounted for 15.6% of all minimally invasive operations within the selected specialties.

rology remained the leading field, representing 61% of all robotic procedures, followed by digestive and general surgery (17%), gynecology (15%), and thoracic surgery (7%). According to official public data, including a parliamentary response published by the French National Assembly (e.g. https://questions.assemblee-nationale.fr/q16/16-9160QE.htm) and institutional communications from the French National Academy of Medicine (e.g. https://www.academie-medecine.fr/wp-content/uploads/2025/10/Communique-Formationchirurgie-robotique-PCRA-82.pdf), the number of robotic surgical systems installed in France has steadily increased in recent years, exceeding 240 systems nationwide by 2023 and confirming the sustained expansion of robotic surgery (Table 1).

**Table 1:** Distribution of robot-assisted surgery stays by specialty and type of institution distribution of hospital stays (%) with at least one CCAM code for robot-assisted surgery

Specialty	2021–Public	2021–Private	2021–Total	2022–Public	2022–Private	2022–Total
Urological surgery	54%	74%	62%	52%	74%	61%
General and digestive surgery	19%	15%	17%	20%	13%	17%
Gynecologic surgery	18%	8%	14%	20%	9%	15%
Thoracic surgery	9%	3%	6%	9%	4%	7%
Total	100%	100%	100%	100%	100%	100%

**Fig. 2:**
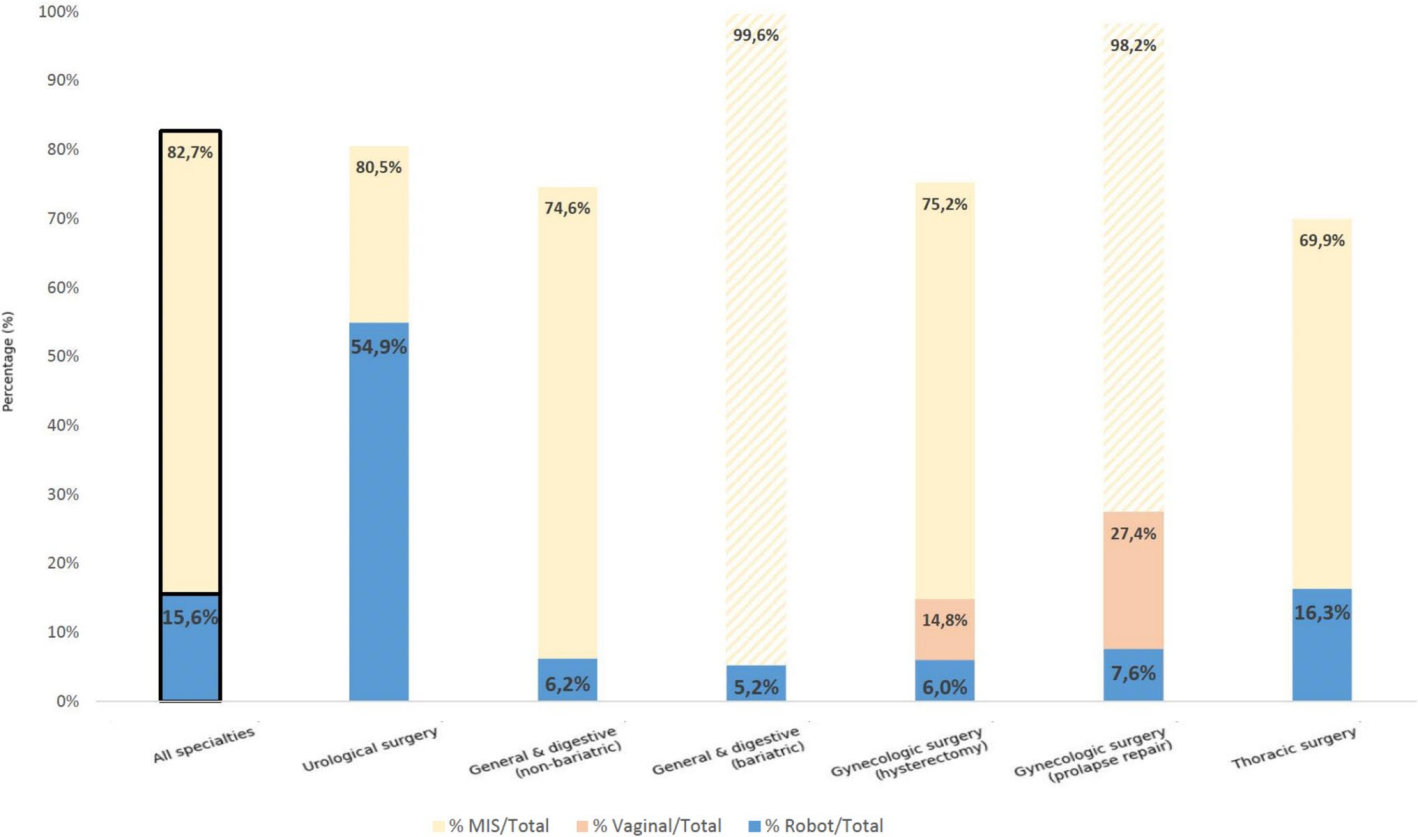
Access rates to different surgical approaches in France in 2022. Percentages correspond to the number of hospital stays including at least one minimally invasive surgery (MIS), robotic, or vaginal procedure in the specialty considered, divided by the total number of hospital stays regardless of surgical approach. Striped bars indicate surgical categories in which open surgery represents a negligible proportion of procedures in the PMSI database

The robotic penetration rate was 54.9% in urology, 13.6% in gynecology, 11.4% in digestive surgery, and 16.3% in thoracic surgery. Procedures such as radical prostatectomy, partial nephrectomy, and hysterectomy were the most frequently performed robotic acts. In contrast, digestive and thoracic applications showed slower growth, reflecting differences in technological adoption and training availability (Fig. 2).

### Regional disparities in access to robotic surgery

**Fig. 3:**
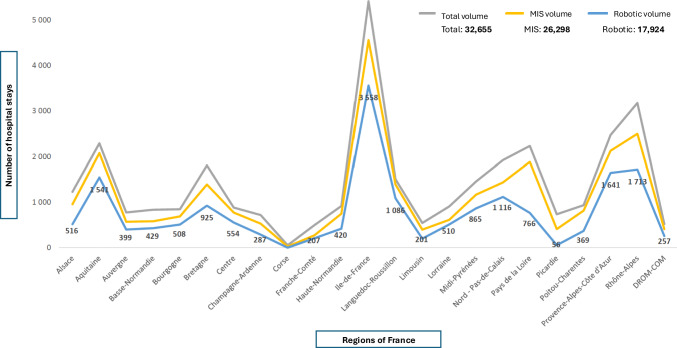
Regional distribution of urologic surgical activity in France according to surgical approach (2022). This figure shows the regional distribution of hospital stays for **urologic surgery** in France (public and private hospitals combined), according to surgical approach (total volume, minimally invasive surgery, and robotic-assisted surgery). Urologic surgery was selected because it represents the highest-volume and most established robotic specialty, allowing robust regional comparisons and clearer visualization of territorial disparities in robotic adoption. Minimally invasive surgery (MIS)

Marked regional inequalities were observed across metropolitan France and overseas territories. The Île-de-France region concentrated more than 27% of all robotic stays, followed by Auvergne-Rhône-Alpes (13%), Provence-Alpes-Côte d’Azur (10%), and Occitanie (9%). Conversely, several regions, including Bourgogne-Franche-Comté, Centre-Val de Loire, and the overseas departments (DROM-COM), showed limited or no recorded robotic activity (Fig. 3).

When adjusted for population size, marked regional disparities persisted across French regions. Using 2022 population data from the French National Institute of Statistics and Economic Studies (INSEE), robotic-assisted surgery rates per 100,000 inhabitants were highest in Île-de-France (approximately 12.5), followed by Auvergne-Rhône-Alpes (approximately 7.1), Provence-Alpes-Côte d’Azur (approximately 6.8), and Occitanie (approximately 5.2). In contrast, Bourgogne-Franche-Comté, Centre-Val de Loire, and overseas departments reported rates below 2 per 100,000 inhabitants (Table 2).

**Table 2:** Regional distribution of robotic-assisted surgery rates per 100,000 inhabitants in France (2022, approximate values)

Administrative region (2022)	Former regions included	Robotic-assisted surgery rate per 100,000 inhabitants
Île-de-France	Île-de-France	12.5
Auvergne–Rhône-Alpes	Auvergne; Rhône-Alpes	7.1
Provence–Alpes–Côte d’Azur	Provence-Alpes-Côte d’Azur	6.8
Occitanie	Languedoc-Roussillon; Midi-Pyrénées	5.2
Nouvelle-Aquitaine	Aquitaine; Limousin; Poitou-Charentes	3-4
Grand Est	Alsace; Champagne-Ardenne; Lorraine	3-4
Hauts-de-France	Nord-Pas-de-Calais; Picardie	2-3
Pays de la Loire	Pays de la Loire	2-3
Normandie	Basse-Normandie; Haute-Normandie	2-3
Bretagne	Bretagne	2-3
Bourgogne–Franche-Comté	Bourgogne; Franche-Comté	<2
Centre-Val de Loire	Centre	<2
Overseas territories (DROM-COM)	Guadeloupe; Martinique; French Guiana; Réunion; Mayotte	<2

The regional robotic penetration rate varied widely, ranging from 0 to 72% depending on the geographical area and surgical specialty. High-density zones corresponded to large university or private referral centers equipped with multiple robotic platforms, whereas peripheral or rural regions remained under-equipped, often relying on conventional laparoscopy.

A north–south gradient was identified, with higher adoption rates in southern and urbanized areas. Public University Hospital accounted for 54% of all robotic stays, private hospitals for 38%, and ESPIC institutions for 8%. The concentration of robotic platforms within a few high-volume centers reflected both economic and organizational constraints, with only 35% of hospitals performing robotic surgery in 2022 despite a steady national increase in installations.

### Comparison with laparoscopic and open approaches

Across all specialties, the open surgery rate remained 17.3%, exceeding 25% in some subspecialties. When robotic, laparoscopic, and open techniques were compared, robotic procedures were associated with shorter hospital stays and lower postoperative ICU utilization.

The mean LOS for robotic procedures was 4.2 ± 2.8 days, representing a reduction of 1.5 days compared with laparoscopic surgery (5.7 ± 3.6 days) and 3.7 days compared with open approaches (7.9 ± 4.8 days) (p < 0.05). The ICU admission rate was 6.3% for robotic cases versus 9.7% for laparoscopic and 14.1% for open surgery. These differences persisted after stratification by specialty and hospital type.

Thirty-day readmission rates were 4.8% for robotic versus 5.6% for laparoscopic and 6.9% for open surgery. Ninety-day readmissions followed the same trend, suggesting lower early postoperative morbidity among robotic cases. However, heterogeneity remained between hospitals, likely influenced by case complexity, patient comorbidities, and learning-curve effects.

### Impact on healthcare organization and system indicators

Robotic surgery was associated with a measurable reduction in resource use. Mean hospital stay decreased by 1.5 days compared with laparoscopy and by 3.7 days compared with open surgery. This reduction translated into an estimated 20 000 bed-days saved nationally in 2022, particularly in high-volume specialties such as urology and gynecology.

Despite these clinical and organizational benefits, the concentration of activity within a limited number of institutions generated pronounced territorial inequities. Several regions performed fewer than 100 robotic procedures per year, raising concerns about equal access to innovative surgical care. The correlation between regional gross domestic product (GDP) per capita and robotic penetration was moderate but significant (r = 0.52; p = 0.01), underscoring the economic component of technological diffusion.

### Reoperation rates by specialty and surgical approach

In urological surgery, 30-day reoperation rates were low across all approaches, remaining below 1% for robotic and laparoscopic procedures (0.7% and 0.8%, respectively), while open surgery was associated with a higher rate of 1.5%. At 90 days, similar patterns were observed, with reoperation rates of 0.9% after robotic surgery, 1.0% after laparoscopic surgery, and 1.6% following open procedures. High-volume robotic centers showed rates comparable to overall robotic surgery at both 30 days (0.7%) and 90 days (1.0%), with the majority of events driven by non-surgical readmissions.

In general and digestive surgery, non-bariatric procedures were associated with higher reoperation rates than urological surgery, particularly after open surgery, which reached 7.4% at 30 days and 8.0% at 90 days. Robotic and laparoscopic approaches demonstrated lower and comparable rates, with 30-day reoperation rates of 3.3% and 3.1%, respectively, and 90-day rates of 4.2% and 3.4%. Bariatric surgery showed consistently low reoperation rates, with laparoscopic procedures accounting for most cases and rates of 1.5% at 30 days and 1.6% at 90 days, while robotic bariatric surgery remained close to 1% at both time points (Table 3).

**Table 3:** 30- and 90-day reoperation rates by specialty and surgical approach

Specialty	Timepoint	Surgical approach	Number of stays	Total reoperation rate (N/%)	Surgical readmission (N/%)	Non-surgical readmission (N/%)
Urological surgery	30 days	Robotic	14 785	100 (0.7%)	<11	90-100
		Laparoscopic	7 766	61 (0.8%)	19 (0.2%)	42 (0.5%)
		Open	5 898	87 (1.5%)	15 (0.3%)	72 (1.2%)
		Robotic - high-volume centers	9 210	66 (0.7%)	<11	56–66
	90 days	Robotic	12 120	111 (0.9%)	<11	101-111
		Laparoscopic	6 368	64 (1.0%)	18 (0.3%)	46 (0.7%)
		Open	4 892	80 (1.6%)	17 (0.3%)	63 (1.3%)
		Robotic - high-volume centers	7 571	72 (1.0%)	<11	62-72
General and digestive surgery - non-bariatric	30 days	Robotic	1 524	51 (3.3%)	39 (2.6%)	12 (0.8%)
		Laparoscopic	27 549	853 (3.1%)	687 (2.5%)	166 (0.6%)
		Open	10 310	761 (7.4%)	572 (5.5%)	189 (1.8%)
		Robotic – high-volume centers	1 037	36 (3.5%)	26–36	<11
	90 days	Robotic	1 250	53 (4.2%)	38 (3.0%)	15 (1.2%)
		Laparoscopic	22 202	757 (3.4%)	593 (2.7%)	164 (0.7%)
		Open	8 469	681 (8.0%)	497 (5.9%)	184 (2.2%)
		Robotic - high-volume centers	843	35 (4.2%)	25-35	<11
General and digestive surgery – bariatric	30 days	Robotic	1 207	15 (1.2%)	11-15	<11
		Laparoscopic	31 918	468 (1.5%)	455 (1.4%)	13 (0.0%)
		Open	141-151	<11	<11	<11
		Robotic - high-volume centers	934	<11	<11	0 (0.0%)
	90 days	Robotic	978	13 (1.3%)	11-13	<11
		Laparoscopic	26 164	420 (1.6%)	401 (1.5%)	19 (0.1%)
		Open	113	<11	<11	<11
		Robotic - high-volume centers	758	<11	<11	0 (0.0%)
Gynecological surgery – hysterectomy	30 days	Robotic	1 135	<11	0 (0.0%)	<11
		Laparoscopic	19 696	26 (0.1%)	<11	16–26
		Vaginal	5 376	11 (0.2%)	<11	<11
		Open	8 978	43 (0.5%)	<11	33–43
		Robotic - high-volume centers	860	<11	0 (0.0%)	<11
	90 days	Robotic	913	<11	0 (0.0%)	<11
		Laparoscopic	15 757	26 (0.2%)	<11	16–26
		Vaginal	4 252	12 (0.3%)	<11	<11
		Open	7 221	52 (0.7%)	<11	42–52
		Robotic - high-volume centers	686	<11	0 (0.0%)	<11
Gynecological surgery – promontofixation	30 days	Robotic	613	0 (0.0%)	0 (0.0%)	0 (0.0%)
		Laparoscopic	11 517	<11	0 (0.0%)	<11
		Vaginal	4 957	13 (0.3%)	11-13	<11
		Open	324	0 (0.0%)	0 (0.0%)	0 (0.0%)
		Robotic - high-volume centers	435	0 (0.0%)	0 (0.0%)	0 (0.0%)
	90 days	Robotic	484	0 (0.0%)	0 (0.0%)	0 (0.0%)
		Laparoscopic	8 963	<11	0 (0.0%)	<11
		Vaginal	3 882	<11	<11	<11
		Open	259	0 (0.0%)	0 (0.0%)	0 (0.0%)
		Robotic - high-volume centers	341	0 (0.0%)	0 (0.0%)	0 (0.0%)

## Discussion

The present national analysis represents, to our knowledge, the first real-world evaluation of robotic-assisted soft tissue surgery in France since the introduction of specific traceability codes in the national PMSI database. The results highlight a sustained growth of robotic activity between 2021 and 2022, with significant disparities across specialties and regions. These findings mirror global trends in robotic surgery, where adoption has accelerated rapidly over the past decade, yet remains unevenly distributed and driven by local economic and institutional factors.

This nationwide PMSI-based analysis provides the first consolidated overview of soft-tissue RAS in France and shows that, despite rapid growth, RAS still accounts for only 15.6% of eligible minimally invasive procedures between 2021 and 2022. During this two-year period, robotic activity increased from 27,691 to 31,221 stays (+ 15.6%), yet open surgery still represented 17.3% of all procedures and exceeded 25% in some subspecialties, highlighting a substantial residual reliance on non-minimally invasive approaches. A proportion of residual open procedures likely reflects emergency surgical indications, which still frequently require an open approach. However, the PMSI database does not allow reliable attribution of surgical approach to emergency status alone, and this should be interpreted with caution.

These findings mirror the recent national report by the French National Academy of Surgery (e.g. https://www.academie-chirurgie.fr/admin/uploads/media/photo/0001/07/cbb1c4f0f4d3c3c1ce88939e7d94bf47b4fd204c.pdf), which also described double-digit growth but moderate absolute volumes and emphasized the “glass ceiling” of conventional laparoscopy in France.

### Clinical and organizational impact

In the present cohort, RAS was associated with shorter hospital stay and lower resource use compared with both laparoscopic and open surgery. The mean LOS after robotic procedures was 4.2 ± 2.8 days, corresponding to a reduction of 1.5 days versus laparoscopy (5.7 ± 3.6 days) and 3.7 days versus open surgery (7.9 ± 4.8 days; p < 0.05). This translated into roughly 20,000 bed days saved in 2022, mainly in urology and gynecology, which is consistent with multiple French PMSI-based analyses demonstrating shorter hospital stays after robotic-assisted surgery compared with laparoscopic and open approaches for various urologic procedures, including radical prostatectomy and nephron-sparing surgery, as well as in high-volume public hospital settings [16–18]. The ICU admission rate was also lower for robotic cases (6.3%) than for laparoscopic (9.7%) and open surgery (14.1%), and 30-day readmission rates followed the same pattern (4.8% vs 5.6% and 6.9%, respectively), suggesting an early morbidity benefit beyond simple shortening of hospital stay.

These observations align with several systematic reviews and meta-analyses reporting shorter LOS and fewer complications with robotic versus open surgery across colorectal, urologic and gynecologic oncology. A recent overview of systematic reviews [19] found that RAS is associated with statistically significant reductions in length of hospital stay compared with both open and laparoscopic procedures, with reductions in LOS frequently in the range of 0.2–2 + days across included indications. However, most economic analyses report higher per-patient costs for RAS than for laparoscopic or open approaches, with incremental costs estimated at approximately $4,600 versus laparoscopy and $3,860 versus open surgery in a cost-minimization model, largely driven by capital equipment, maintenance, and supply costs. The present nationwide data corroborate this gradient: most of the gain is observed when RAS replaces open surgery, whereas differences versus laparoscopy are smaller and may reflect better ergonomics, tremor filtration and easier access to deep pelvic or thoracic regions rather than a fundamentally different operative strategy.

Reoperation rates in this study were low overall and comparable between robotic and laparoscopic approaches, while consistently higher after open surgery, particularly in general and digestive surgery. In urology, 30-day reoperations remained below 1% after both robotic (0.7%) and laparoscopic (0.8%) procedures, versus 1.5% after open surgery, with similar patterns at 90 days (0.9%, 1.0% and 1.6%, respectively). Although 90-day readmission rates were captured at a national level, the PMSI database does not allow reliable attribution of readmissions to specific clinical causes. These readmissions likely reflect a heterogeneous mix of postoperative complications, delayed surgical management, and non–procedure-related admissions, and should therefore be interpreted as global outcome indicators rather than cause-specific events.

Non-bariatric digestive procedures showed higher absolute reoperation rates, up to 7.4% at 30 days after open surgery, compared with 3.3% and 3.1% after robotic and laparoscopic approaches, respectively. These figures are in line with international data indicating that RAS achieves at least equivalent, and often slightly lower, early complication and reoperation rates than laparoscopy, while clearly outperforming open surgery for several digestive and colorectal indications [20].​​ The slightly higher 90-day reoperation rate observed after robotic general and digestive surgery should be interpreted with caution. Robotic approaches are often used for more complex cases and may be influenced by learning-curve effects, which cannot be adjusted for in the PMSI database. Given the small absolute difference observed and the consistently lower rates compared with open surgery, this finding is unlikely to be clinically meaningful.

### Regional disparities and equity of access

Beyond clinical outcomes, our study provides a detailed quantification of territorial inequalities in access to RAS in France. While absolute numbers show that Île-de-France accounted for more than 27% of robotic stays, followed by Auvergne Rhône-Alpes (13%), Provence Alpes Côte d’Azur (10%), and Occitanie (9%), normalizing these figures by regional population reveals persistent disparities. Using population data from French National Institute of Statistics and Economic Studies (data from 2022, available online: https://www.insee.fr/fr/statistiques), we calculated RAS procedures per 100,000 inhabitants: Île-de-France (~ 12.5/100,000), Auvergne Rhône-Alpes (~ 7.1/100,000), Provence Alpes Côte d’Azur (~ 6.8/100,000), Occitanie (~ 5.2/100,000), whereas Bourgogne-Franche-Comté, Centre-Val de Loire, and the overseas departments reported densities below 2/100,000. Importantly, normalization of robotic-assisted surgery activity to population size did not eliminate territorial disparities, indicating that these inequalities are unlikely to be explained by demographic factors alone and more likely reflect structural and organizational determinants. These findings indicate that the unequal distribution of robotic surgery is not merely a reflection of regional population size, but also reflects differences in infrastructure, availability of robotic platforms, and concentration of specialized surgical centers. Similar patterns of regional concentration have been previously reported in French urologic surgery, with robotic prostatectomy and partial nephrectomy predominantly performed in high-volume urban centers [17, 19, 21].

Highlighting RAS rates relative to population strengthens the interpretation of uneven access and underscores the need for targeted policies and investment to improve equitable distribution of robotic surgical resources across French regions.

These findings echo reports from other European settings, where robotic systems tend to cluster in wealthier or academic regions, raising concerns about “two-tier” access to advanced minimally invasive surgery. In gynecologic oncology, for example, Scandinavian and UK initiatives have deliberately centralized robotic surgery in accredited cancer centers to standardize indications and mitigate unwarranted regional variation [6, 7, 22, 23].

In France, the persistence of high residual open surgery rates for procedures widely recommended to be performed via minimally invasive approaches reaching 19.5% in urology, 25.4% in non-bariatric digestive surgery, 24.8% in hysterectomy and 30.1% in thoracic surgery, with regional peaks exceeding 40–80% was also derived from analyses of the national PMSI database. These findings suggest that access to both laparoscopy and RAS remains uneven more than three decades after their introduction. Importantly, regions with limited or no access to robotic platforms also tended to exhibit higher proportions of open procedures, whereas regions with greater robotic penetration showed lower residual open surgery rates. Although this association was assessed descriptively and not adjusted for case-mix or hospital volume, it supports the hypothesis that structural and technological disparities contribute to persistent regional differences in surgical practice. Similar patterns have been reported in other European healthcare systems: a recent nationwide study from England demonstrated that regional deprivation and resource availability were strongly associated with poorer access to laparoscopic and robotic colorectal surgery, highlighting how unequal diffusion of minimally invasive technologies can sustain geographic disparities in surgical care [21]. Although patient-level socioeconomic data were not available in the PMSI, social deprivation likely contributes to the observed territorial inequalities in access to robotic-assisted surgery, acting through regional differences in healthcare infrastructure, hospital volume, and technological resources.

From a public-health perspective, this heterogeneity challenges the principles of relevance and equity of care that underpin the French universal health system [24].

### Implications for policy, training and “Surgery 4.0”

Taken together, these results support the view that RAS should be considered not only as a surgical tool but as a digital platform embedded in the broader transition toward “Surgery 4.0” and 5P medicine. Several expert bodies, including the Royal College of Surgeons of England and French professional societies, now recommend coupling the deployment of robotic platforms with structured training curricula, minimum-volume requirements and systematic collection of outcome indicators [6]. The present study demonstrates that PMSI can be leveraged at national scale to monitor RAS activity, track temporal trends and compare outcomes between approaches, but also reveals the current absence of a coordinated national policy for robotic allocation, training and evaluation.

A programmed, data-driven robotization strategy, similar to what has been implemented for certain gynecologic and colorectal cancer pathways in Denmark and other European countries, could help overcome the “laparoscopy ceiling” and reduce unwarranted reliance on open surgery [7]. Such a strategy would likely include: (i) regional planning of robotic platforms to ensure a minimum coverage of high-volume indications; (ii) designation of reference centers with sufficient caseload to maintain expertise; (iii) integration of RAS outcomes into national quality indicators; and (iv) economic models that re-invest LOS-related savings into capital and maintenance costs. Early cost-effectiveness studies suggest that, when LOS reduction reaches 1–2 days and complication rates decrease, RAS can approach cost-neutrality or even generate net savings at system level, particularly for complex oncologic procedures [22].

### Strengths, limitations and future research

The main strengths of this study are its exhaustive national coverage, the use of mandatory CCAM traceability codes for RAS and the inclusion of multiple specialties, which allow robust estimates of volumes, outcomes and regional disparities. Unlike small single-center series, this analysis provides real-world evidence on more than 58,000 robotic stays over two years and quantifies their impact on key system indicators such as LOS, ICU use and readmissions. However, several limitations must be acknowledged. First, PMSI is an administrative database that lacks detailed clinical variables (tumor staging, BMI, frailty indices, operative time, intraoperative complications), which restricts risk adjustment and may confound comparisons between approaches. Although a substantial proportion of procedures were performed for malignant indications, this study does not assess oncologic outcomes such as tumor staging, recurrence, or survival. The term “oncologic surgery” is used solely to describe the clinical context of the procedures, and analyses were restricted to perioperative and organizational outcomes captured in the PMSI database.

Second, the relatively short period since the introduction of robotic traceability (mandatory since March 2020) limits long-term trend analysis and makes some subgroup comparisons underpowered, particularly for thoracic and bariatric indications.

Third, coding practices may vary between institutions, potentially leading to under- or over-estimation of RAS activity or residual open surgery in certain regions. Finally, cost data and patient-reported outcomes are not available in PMSI, whereas they are crucial to fully assess the value of RAS in a 5P-medicine framework. Future work should therefore focus on linking PMSI to clinical registries and robotic platform data (kinematics, workflow metrics), enabling more refined analyses of learning curves, oncologic outcomes and quality of life, as well as on developing prospective, indication-specific studies that compare optimized laparoscopic and robotic programs within standardized care pathways [25].

While centralization of robotic surgery in high-volume centers may support quality and expertise, it may also generate bottlenecks and longer waiting times in publicly funded healthcare systems. Alternative organizational models, including targeted deployment of robotic platforms in underserved or geographically isolated regions, warrant further evaluation. Future studies using AI-based modeling could compare centralized versus strategically distributed approaches by integrating travel distance, waiting times, and regional case volume, with the aim of optimizing both efficiency and equity of access.

## Conclusion

This nationwide PMSI-based study provides the first real-world assessment of robotic-assisted soft tissue surgery in France. Robotic activity increased substantially between 2021 and 2022, confirming its integration into contemporary surgical practice, but with marked regional and specialty-related disparities.

Robotic approaches were associated with shorter hospital stays, reduced ICU utilization, and lower readmission rates compared with laparoscopic and open surgery, supporting their clinical and organizational value. However, unequal access and heterogeneous implementation highlight the need for coordinated oversight.

A national strategy integrating investment planning, standardized training, and data-driven evaluation is now essential to ensure equitable and sustainable deployment. Beyond a technical innovation, robotic surgery represents a key enabler of Surgery 4.0, supporting the transition toward predictive, personalized, and evidence-based surgical care.

## Data Availability

No datasets were generated or analysed during the current study.
